# Factors associated with oocyte recovery rates during in-vitro fertilization among Nigerian women

**DOI:** 10.11604/pamj.2024.47.190.38674

**Published:** 2024-04-16

**Authors:** Amina Mohammed-Durosinlorun, Ibrahim Wada

**Affiliations:** 1 Department of Obstetrics and Gynaecology, Faculty of Clinical Sciences, College of Medicine, Kaduna State University/Barau Dikko Teaching Hospital, Kaduna, Nigeria,; 2 Nisa Premier Hospital/Institute of Medical Sciences, Abuja, Nigeria

**Keywords:** Oocyte recovery rates (ORR), in-vitro fertilization, Nigerian women

## Abstract

**Introduction:**

the availability of oocytes is fundamental to in vitro fertilization (IVF). The factors associated with optimal or suboptimal oocyte recovery rates (ORR) in low-resource settings are not well known. This study aimed to determine the factors associated with ORR by comparing demographic and IVF cycle data of women undergoing IVF in our Centre.

**Methods:**

this was a prospective study of 110 infertile women undergoing IVF at Nisa Premier Hospital, Abuja Nigeria, from October 2020 to September 2021. All women had reached the stage of oocyte retrieval or further, after receiving ovarian stimulation with our routine protocols. Treatment was monitored by serial transvaginal ultrasonography. The oocyte retrieval procedures were performed under conscious sedation, 36 hours after the ovulatory trigger. Optimal ORR was when eggs were obtained from at least 80% of follicles punctured. Sub-optimal ORR was when it was less than 80%. Data analyses utilized SPSS statistical software and a p-value of < 0.05 was considered significant.

**Results:**

the mean age of all women was 34.1±4.9 years. Sixty-nine women (62.7%) had sub-optimal ORR while 41 (37.3%) had optimal ORR. Six women (5.5%) had no oocytes retrieved. Significantly more women with sub-optimal ORR were obese (70.6 vs 29.4%) and had higher follicle-stimulating hormone (FSH) levels (8.11 vs 6.34 miu/ml), p-value- 0.039. Women with sub-optimal ORR had higher mean prolactin levels (17.10 ± 13.93 miu/ml) than women with optimal ORR 11.43 ± 6.65 miu/ml), p-value- 0.019). Significantly more oocytes (5.99 vs 10.37, p-value 0.001), and MII oocytes (5.78 vs 7.56, p-value 0.035) were retrieved in women with optimal than sub-optimal ORR. The duration of stimulation, total amounts of gonadotropins administered, and fertilized oocytes were not significantly different among both groups (p-value >0.05).

**Conclusion:**

this study has shown the factors associated with ORR in our setting to be basal FSH, prolactin, and obesity.

## Introduction

Infertility occurs if there is a failure to achieve pregnancy after one year of regular, unprotected sexual intercourse [1,2]. It is of significant public health concern [3,4] with an estimated worldwide prevalence of 8-12% [2]. Prevalence is much higher in sub-Saharan Africa [5-8] Nigeria is included in the African demographic infertility belt, and paradoxically has both high fertility rates, coexisting with high infertility rates [3]. Some studies (mostly facility-based) have quoted the prevalence of infertility within Nigeria, as 26.8% in Lagos [9], 15.4% in Abakaliki [10], 15.7% in Sokoto [11], and 23.9% in Bauchi [12]. The prevalence may be on the rise as more women delay childbearing for education, work, and other reasons [3,13].

The adverse effects of infertility especially by women, are exaggerated in our environment due to cultural expectations and fertility preferences [14]. These negative effects include social isolation, neglect, abuse, physical violence, marital discord, stigmatization, and depression [15]. Overall, the quality of life is reduced. In trying to overcome their infertility, couples may face long, frustrating, and expensive treatments without full guarantees of success [16]. The aetiology of infertility also varies widely with contributions from both the male (30-40%), and the female partner (30-40%), and combined/sometimes it is unexplained (20-40%) [17]. Among the various causes of infertility, tubal factor infertility is more common in this environment, related to complications of sexually transmitted, postabortal, and postpartum infections [18-24]. This form of infertility is best treated with assisted reproductive techniques (ART) [21,24]. Assisted reproductive techniques may also be the last option for couples with infertility if other treatments have failed. In-vitro fertilization (IVF) is one of the most widely used interventions for infertility [25]. One Nigerian study estimated that 24.5% of the studied population would require and benefit from ART [26].

Assisted reproductive techniques involve in vitro manipulation of the gametes. Pioneering work in Cambridge by Steptoe and Edwards lead to the birth of Louise Brown, the first IVF baby. The field of ART has since grown, and a lot of new drugs and techniques introduced to improve success. By the end of 2013, five million IVF babies were born worldwide [27]. In Nigeria, over 40,000 babies have been born through IVF [28]. In Nigeria, the early work done by the likes of Ashiru and Giwa-Osagie in Lagos, Wada in Abuja, Orhue in Benin, and Ikechebelu in Nnewi has made ART widely available in some parts of Nigeria but is still inadequate to meet demands [28-30]

Oocyte retrieval is a critical step in the ART/IVF process as it´s the only way to make the oocytes available for external fertilization. Furthermore, success at this stage is important to boost the morale of the couples. In the early days when IVF was performed using natural cycles, the target was the retrieval of one oocyte, thus the success rate at the time was low with a live birth rate (LBR) of 9.6% [31]. Subsequently, ovarian stimulation with drugs like clomiphene and gonadotropins led to multiple oocytes being retrieved and higher success rates [32,33]. In addition, excess eggs and embryos generated can be frozen and used later or even donated to research or use by other couples.

The reported oocyte recovery rate in natural cycles is 80% [34]. but with ovarian stimulation, the number of oocytes retrieved can be much lower than the number of follicles observed on ultrasonography. This can be because some follicles may develop but do not contain any oocytes [35]. The fact that at the end of an IVF stimulated cycle, few or no oocytes may be retrieved can be a disaster, more so if serial ultrasound scans had shown the presence of several promising follicles. The couple (and health care personnel) would have undergone a lot of psychological stress, sacrificed a lot of time, and spent a lot of resources without obtaining their objective [36,37].

A rare and controversial “empty follicle syndrome” (EFS) was first described by Coulam *et al*. in 1986 [38]. The reason for unsuccessful or sub-optimal oocyte retrieval was not clear. It has however been attributed to iatrogenic and technical/drug-related causes (false EFS) [39,40], dysfunctional folliculogenesis with early oocyte atresia, genetic cause, ovarian aging, and poor ovarian response (genuine EFS) [40-44]. A borderline form of EFS has also been described as cases where very few mature or immature oocytes are retrieved from the aspiration of several follicles after a satisfactory ovarian stimulation regimen [45,46].

Despite developments of ART, 35-40% of couples remain childless after treatment hence science still has a lot to understand and develop to continuously improve success rates [47]. This study aims to investigate the factors affecting the disparity between follicles seen, and oocytes retrieved in stimulated IVF cycles among infertile Nigerian women. Fertility research is generally sparse in this environment despite the huge burden of infertility, cultural aversion to adoption or third-party treatments, and the high cost of ART, which makes the technology inaccessible to most people who need it in Nigeria [48,49]. So, sub-optimal oocyte retrieval constitutes an additional burden because it can potentially halt treatment. Very few studies have examined the disparity between follicles seen and oocytes retrieved during IVF. Most studies done previously have been retrospective or studied other related aspects like follicular size or volume [50-52]. There is a dearth of information from low-resource settings on ORR and it is expected that findings in this study would contribute to the body of knowledge on this subject. This may ultimately help counsel infertile couples planning to start IVF, stimulate further research, and direct the development of treatments to maximize oocyte retrieval, leading ultimately to improved success rates.

## Methods

**Design:** this was a prospective study.

**Setting:** the study was carried out at the Nisa Premier Hospital (Nisa) located in Jabi, Abuja; Federal Capital Territory (FCT) Nigeria. The hospital has a capacity for 60 adult in-patient beds and 40 baby cots and provides a wide range of services in different specialties: general outpatient services, family medicine, obstetrics and gynaecology, surgery, and paediatrics among others [53]. The hospital does an average of 400-500 fresh/frozen IVF/ICSI cycles per year.

**Study population:** infertile women were seen at the Nisa Premier Hospital from 1^st^ October 2020 to 30^th^ September 2021), Abuja with the following eligibility criteria.

**Inclusion criteria:** all infertile women ≥ 18 years coming for IVF/ICSI cycles that have been selected for controlled ovarian hyperstimulation during the study period (irrespective of whether they have had previous stimulation or not), women with both ovaries present and visible on transvaginal ultrasound during follicular tracking, women whose oocyte retrieval (OCR) was done via the transvaginal route.

**Exclusion criteria:** women not willing to participate in the study, or whose cycles were canceled before oocyte retrieval, women with previous ovarian surgery, chemotherapy, or pelvic radiotherapy. Menopausal women undergoing third-party programs were excluded. Cycles with significant technical difficulties or complications during OCR leading to incomplete retrievals were excluded.

**Sampling:** a convenient sample of all patients meeting the study criteria over one year as stated earlier was used.

**Procedure:** all women who gave consent to participate in the study were recruited consecutively until the sample size was attained. After a proper history was taken and a physical examination done, routine infertility investigations were done. Routine hormonal measurements were done on day 3 (2-5) of the menstrual cycle. Ovarian stimulation was done using standardized agonist or antagonist protocols. Protocols used and starting doses of drugs vary depending on factors such as age, hormonal levels, previous response, and risk of ovarian hyperstimulation. The dose was adjusted appropriately based on patients´ responses. Usually, Buserellin was used for agonist cycles, cetrorelix (Cetrotide®, Merck Serono, Darmstadt, Germany) for antagonist protocols, and various gonadotrophins (Menopur- Ferring Pharmaceuticals, Copenhagen, Denmark; or Gonal-F, Merck Serono, Darmstadt, Germany).

Follicular tracking was done by experienced fertility doctors using multiple transvaginal ultrasonographic scans. Ultrasound were done using Voluson P8 scanning machines (GE Healthcare Technology). Serum oestradiol was also used to monitor the cycle. Human chorionic gonadotropin (hCG) or Buserelin trigger was given depending on the protocol when a leading follicle measured ≥18 mm in diameter. Oocyte retrieval was carried out 35-36 hours after trigger by ultrasound-guided transvaginal puncture using a single lumen needle (Kitazato) under sedation. Routine embryo transfer was done on days 2-5 as indicated. Sometimes cryopreservation was required. Routine oocyte/embryo culture was done by a trained embryologist and 1 to 3 embryos were transferred on days 3-5 as indicated. Luteal support was usually initiated 1 day after oocyte pick-up. Clients were followed up for a minimum period of 2 weeks after embryo transfer and records were retrieved. Serum pregnancy test was done 10-12 days after embryo transfer (ultrasound 5-6 weeks later when possible).

**Data collection:** a data extraction form was used to record important demographic information and clinical correlates for each patient including demographic information, history about infertility and diagnosis, cycle/treatment characteristics including induction protocol, relevant hormonal/laboratory and TVUS (transvaginal ultrasonography) data, OCR data, and oocyte characteristics. Patient records were checked later to determine pregnancy outcomes or complications.

**Data analysis:** the primary outcome measure for our study was oocyte recovery rate (ORR) in stimulated IVF cycles among pregnant women. ORR was defined as the number of oocytes retrieved/number of follicles (seen on ultrasound at the end of ovarian stimulation and before oocyte retrieval) x 100. The ORR was considered to be optimal if it was ≥80%, and sub-optimal if it was <80%. Other secondary outcomes included: empty follicle syndrome (EFS) defined as the complete failure to recover any oocytes during OCR, with and without a detectable serum β-hCG on the day of retrieval, Oocyte quality which was classified as good (MII with good polar body) or poor (MI, GV- germinal vesicle, EZ- empty zona or atretic). Fertilized eggs were confirmed by the presence of 2 pronuclei), and a confirmed pregnancy. A chemical pregnancy was defined as a serum hCG >10IU/l done 10-12 days after embryo transfer while a clinical pregnancy was defined as finding a gestational sac, foetal pole, and foetal heartbeat on ultrasound at 5-6 weeks of gestation.

Statistical analysis was done using IBM SPPS Statistics 22 (Armonk, NY: IBM Corp). Baseline characteristics were summarized using simple frequency tables. To investigate the factors that may affect ORR in stimulated IV cycles among infertile women, dependent variables (demographic and reproductive characteristics such as age, ethnicity, parity, previous miscarriage, BMI, menstrual cycle characteristics, infertility, and lifestyle characteristics) were compared with the independent variable (optimal and sub-optimal ORR) using Chi-square test/Fisher's exact test for categorical variables and t-test to compare group means of quantitative data. A p-value of < 0.05 was considered to be of statistical significance.

**Ethical approval:** ethical approval was obtained from the Nisa Premier Hospital ethical committee and informed consent from patients. Most of the information, procedures, and investigations were already part of the routine work-up that the patients undergo for IVF. So, there was no additional monetary or psychological cost to patients. If patients declined consent, the quality of care they received was not affected. All patient identifiers were removed to maintain confidentiality.

## Results

A total of 110 women were studied. Their baseline characteristics are shown in Table 1. Their mean age was 34.1±4.9 years, the median age of 35 years and, a range of 22 to 45 years. Most study participants were Hausa (49, 45.5%), multiparous (59, 53.6%), had no previous miscarriages (72, 65.5%), overweight or obese (74, 67.3%), had normal menstrual characteristics, had secondary infertility (59, 53.6%), with infertility for 1-5 years (72, 65.4%), with female factor infertility (69,62.7%), and received no previous fertility treatment (78. 70.9%). This is shown in Table 1. Sixty-nine women (62.7%) had sub-optimal ORR (<80%) while 41 women (37.3%) had optimal ORR (≥80%). Six women (5.5%) had no oocytes retrieved. The client's BMI (body mass index), the pattern of menstruation (regularity, length, and flow), and trigger type were significantly associated with ORR (P-value <0.05) as shown in Table 2. When comparing means (Table 3), there were significant differences in FSH levels, prolactin levels, total number of oocytes retrieved, and number of MII (mature) oocytes. Though number of oocytes that got fertilized was slightly higher in the optimal ORR group compared to the sub-optimal group, the difference was not statistically significant.

**Table 1:** baseline characteristics of participants

Characteristics		Frequency	Percent
**Age**	<30	22	20
	30-34	30	27.3
	35-39	43	39.1
	≥ 40	15	13.6
**Ethnicity**	Hausa	49	45.5
	Igbo	27	24.5
	Yoruba	15	13.6
	Others	19	17.3
**Parity**	0	51	46.4
	1-4	54	49.1
	≥ 5	5	4.5
**Previous miscarriage**	No	72	65.5
	Yes	38	34.5
**Body mass index (BMI)**	< 18.5 (underweight)	0	0
	18.5 - <25 (normal weight)	6	5.5
	25 - <30 (overweight)	40	36.4
	≥ 30 (obese)	34	30.9
	Missing	30	27.2
**Menstrual characteristics**	**Age at menarche (years)**		
	10 – 16	87	79.1
	≥ 17	5	4.5
	Missing	18	16.4
**Cycle regularity**	Regular	89	80.9
	Irregular	15	13.6
	Missing	6	5.5
**Cycle length (days)**	21– 35	101	91.8
	>35	2	1.8
	Missing	7	6.4
**Flow duration (days)**	2-8	99	90
	≥ 9	5	4.5
	Missing	6	5.5
**Type of infertility**	Primary	51	46.4
	Secondary	59	53.6
**Duration of infertility (years)**	< 1	10	9.1
	1-5	72	65.4
	6-10	18	16.4
	>10	10	9.1
**Cause of infertility**	Male factor	10	9.1
	Female factor	69	62.7
	Combined	23	20.9
	Unexplained	8	7.3
**Lifestyle>**	**Drinks**		
	No	104	94.5
	Yes	6	5.5
**Smokes**	No	108	98.2
	Yes	2	1.8
**Previous fertility treatment**	No	78	70.9
	Yes	32	29.1

ORR: oocyte recovery rate

**Table 2:** association between demographic, reproductive, and other variables with ORR

		ORR		Test statistic and p- value
**Variables**	**Age**	<80%	≥ 80%	
	<30	15(68.2)	7(31.8)	
	30-34	16(53.3)	14(46.7)	X2- 9.868, df-6, p-value-0.140
	35-39	25(58.1)	18(41.9)	
	≥ 40	13(86.7)	2(13.3)	
**Ethnicity**	Hausa	31(63.3)	18(36.7)	X2- 1.113, df-3, p-value-0.774
	Igbo	18(66.7)	9(33.3)	
	Yoruba	10(66.7)	3(33.3)	
	Others	10(52.6)	9(47.4)	
**Parity**	0	32(62.7)	19(37.3)	Likelihood ratio- 0.761, df-2, p-value-0.684
	1-4	33(61.1)	21(38.9)	
	≥ 5	4(80)	1(20.0)	
**Previous miscarriage**	No	47(65.3)	25(22.7)	X2- 0.580, df-1, p-value-0.446
	Yes	22(57.9)	16(42.1)	
**Body mass index (BMI)**	18.5 - <25 (normal)	6(100.0)	0(0)	Likelihood ratio- 8.359, df-3, p-value-0.039
	25 - <30 (overweight)	21(52.5)	19(47.5)	
	≥ 30 (obese)	24(70.6)	10(29.4)	
	Missing	18(60.0)	12(40)	
**Menstruation**	**Age at menarche (years)**			Likelihood ratio- 5.472, df-2, p-value-0.065
	10 – 16	58(66.7)	29(33.3)	
	≥ 17	4(80)	1(20.0)	
	Missing	7(38.9)	11(61.1)	
**Cycle regularity**	Regular	58(65.2)	31(34.8)	Likelihood ratio-12.830, df-2, p-value-0.002
	Irregular	11(73.3)	4(26.7)	
	Missing	0(0)	6(100)	
**Cycle length (days)**	21– 35	66(65.3)	35(34.7)	Likelihood ratio- 9.199, df-2, p-value-0.010
	>35	2(100.0)	0(0)	
	Missing	1(14.3)	6(85.7)	
**Flow duration (days)**	2-8	68(68.7)	31(31.3)	Likelihood ratio-17.209, df-2, p-value-0.000
	≥ 9	1(20.0)	4(80.0)	
	Missing	0(0)	6(100)	
**Type of infertility**	Primary	32(62.7)	19(37.3)	Likelihood ratio- 0.000, df-1, p-value-0.997
	Secondary	37(62.7)	22(37.3)	
**Duration of infertility (years)**	< 1	5(50.0)	5(50.0)	Likelihood ratio- 4.926, df-3, p-value-0.177
	1-5	45(62.5)	27(37.5)	
	6-10	10(55.6)	8(44.4)	
	>10	9(90.0)	1(10.0)	
**Cause of infertility**	Male factor	6(60.0)	4(40.0)	Likelihood ratio- 3.416, df-3, -value-0.332
	Female factor	43(62.3)	26(37.7)	
	Combined	17(73.9)	6(26.1)	
	Unexplained	3(37.5)	5(62.5)	
**Lifestyle>**	**Drinks**			Likelihood ratio- 2.251, df-, p-value-0.133
	No	67(64.4)	37(35.6)	
	Yes	2(33.3)	4(66.7)	
**Smokes**	No	69(63.9)	39(36.1)	Likelihood ratio- 4.010, df-1, p-value-0.045
	Yes	0(0)	2(100.0)	
**Previous fertility treatment**	No	46(59.0)	32(41.0)	Likelihood ratio- 1.615, df-1, p-value-0.204
	Yes	23(79.1)	9(28.1)	
**Stimulation protocol used**	Agonist	33(63.5)	19(36.5)	X2- 0.023, df-1, p-value-0.880
	Antagonist	36(62.1)	22(37.9)	
**Trigger type**	Buserelin	11(44)	14(56)	Likelihood ratio- 12.124, df-2, p-value-0.002
	HCG	57(72.2)	22(27.8)	
	Missing	1(16.7)	5(83.3)	
**Serum pregnancy test**	Negative	20(60.6)	13(39.4)	X2- 8.756, df-2, p-value-0.013
	Positive	13(43.3)	17(56.7)	
	Missing	36(76.6)	11(23.4)	

(%): row percentages, ORR: oocyte recovery rate, X^2^: Chi-square, df: degree of freedom

**Table 3:** comparison of variable means between optimal and sub-optimal ORR groups

Variable	ORR groups		P-value
	Sub-optimal ORR (<80%)	Optimal ORR (≥ 80%)	
Mean age (years)	34.55 ± 5.29 (69)	33.37 ± 4.15 (41)	0.195
Total gonadotrophin use (I.U)	3291.41 ± 1390.38 (68)	3377.08 ± 1172.70 (36)	0.848
Duration of stimulation (days)	10.66 ± 1.3 (68)	10.64 ± 0.96 (36)	0.926
Peak oestradiol (E2) (pg/ml)	3218.63 ± 4897 (66)	3897.99 ± 6611 (36)	0.559
AMH (ng/ml)	2.48 ± 2.51 (5)	3.96 ± 2.39 (11)	0.278
FSH (miu/ml)	8.11 ± 4.46 (68)	6.34 ± 3.70 (39)	0.039
LH (miu/ml)	3.67 ± 2.39 (68)	3.92 ± 2.57 (40)	0.614
Prolactin (miu/ml)	17.10 ± 13.93 (68)	11.43 ± 6.65 (39)	0.019
Progesterone (ng/ml)	4.04 ± 3.42 (35)	3.27 ± 2.90 (19)	0.411
Basal AFC	11.31 ± 5.94 (68)	11.46 ± 6.30 (41)	0.898
Total follicles (trigger day)	10.32 ± 6.46 (69)	10.83 ± 6.67 (41)	0.093
Number retrieved oocytes	5.99 ± 4.89 (69)	10.37 ± 6.32(41)	0.001
Oocytes >14-16mm	7.96 ± 4.95 (69)	8.20 ± 5.05 (41)	0.809
MII oocytes	5.78 ± 3.86 (58)	7.56 ± 4.42 (41)	0.035
Number fertilized	5.30 ± 3.82 (57)	6.71 ± 3.96 (41)	0.079

**Note:** values are mean ± standard deviation (number of clients), AMH: anti mullerian hormone, FSH: follicle stimulating hormone, LH: luteinizing hormone, AFC: antral follicular count, ORR: oocyte recovery rate

Due to loss at follow-up, there was inadequate data on clinical outcomes to compare among the groups. There were 63 fresh cycles while the others had their cycle either canceled or converted to a frozen cycle (and some are yet to return) due to several reasons; no oocytes retrieved, only immature oocytes retrieved, progesterone was high, arrested growth especially after biopsy, high risk for ovarian hyperstimulation syndrome and spouse unable to make sperm available. Available data however showed 30 biochemical pregnancies/positive pregnancy tests (33 negative pregnancy tests, 47 missing data on pregnancy tests), 19 clinical pregnancies (11 singletons, 7 twins, and 1 triplet), 6 miscarriages, 1 live birth, and no cases of moderate or severe ovarian hyperstimulation syndrome (OHSS).

## Discussion

During IVF, several studies agree that for each additional egg retrieved, the chances of having a live birth are increased, to the optimum of 6-15 eggs retrieved [54-56]. This study aimed to determine factors associated will non-retrieval of oocytes from follicles seen on ultrasound during ovarian stimulation leading to optimal or sub-optimal ORR. Very few researchers have studied this, especially in our environment.

In our study, women who were overweight and obese had more sub-optimal, than optimal ORR (p-value <0.05) (Table 2). Extremes of weight have traditionally been associated with poor fertility outcomes though studies give controversial results. One cohort study of 342 treatment cycles reported sub-optimal outcomes including significantly fewer mature (MII) oocytes in overweight women, but this was only in the first treatment and suggested an effect modification related to the treatment cycle number [57]. Other studies however showed that being overweight or obese did not adversely affect ovarian function [58]. It may seem wise to continue to advise women to be of optimal weight before embarking on IVF treatment.

The menstrual pattern was significantly associated with ORR (Table 2). This is probably because a regular menstrual cycle is assumed to be a reflection of normal levels of reproductive and regulatory hormones that would result in ovulation [59]. Women with polycystic ovarian syndrome (PCOS) are also known to have irregular menstrual cycles. In a study of 1834 women with PCOS undergoing IVF, women with amenorrhoea had significantly higher incidence of adverse pregnancy outcomes like abortion, preterm births, gestational diabetes, hypertension, premature rupture of membranes and macrosomia [60]. A study of fertility and menstrual patterns in 470 women showed that conception following menstrual cycles of abnormal length were more likely to be aborted, while cycle flow of more than 5 days was associated with fewer abortions [59]. A cycle flow of 5 days also had the highest fecundity [59]. Perhaps the association of menstrual patterns and ORR may be due to the underlying cause of abnormal menstruation patterns such as PCOS, underlying hormonal dysfunction, or some unexplained cause requiring further study.

In our study, the mean FSH levels were significantly associated with ORR, higher in the sub-optimal group (Table 3). This is similar to findings from a Pakistani study, where a higher basal FSH follicle was significantly correlated with a higher incidence of EFS [61]. Women with diminished ovarian reserve have higher FSH to try and compensate, and generally have poorer response to ovarian stimulation and IVF outcomes, especially if FSH levels are above 18 IU/L [62]. The trio of BMI, FSH and abnormal menstrual patterns found in this study to be related to ORR may not be unrelated to a PCOS [63].

Prolactin levels were also associated with ORR in our study. Hyperprolactinaemia (serum prolactin > 25ng/ml) is a known cause of anovulation and infertility. Prolactin may however have a role in oocyte maturation and embryonic development, as some studies found that higher basal prolactin levels (> 16.05 ng/mL) were associated with larger numbers of mature oocytes and good-quality embryos. This contradicts our finding as the mean levels of prolactin were higher in the sub-optimal ORR group. Both groups in our study however had prolactin levels ≤ 50 ng/mL, and some studies have suggested that prolactin levels ≤ 50 ng/mL may not require any treatment [64,65].

In this study trigger type was significantly associated with ORR, with more women using HCG having optimal ORR than Buserelin (Table 2). The natural LH surge is longer (48 hours) as compared to the GnRH analogue induced LH surge (28-32) but the implications of this are unclear, and some studies show that choice of trigger may not increase the incidence of EFS [66,67]. An additional benefit of GnRH analogue for the trigger is that it also induces an FSH surge, keeping gap junctions open between the oocyte and cumulus cells, and activates plasminogen facilitating follicle detachment [68]. Human chorionic gonadotropin does not provide this additional FSH surge, and the use of dual triggers has been suggested [65]. Human chorionic gonadotrophin or GnRH (gonadotrophin-releasing hormone) analogues can be used as natural LH (luteinising hormone) substitutes [69] to trigger the final maturation of oocytes [70,71]. They also induce the meiotic reactivation of the oocyte and enhance the detachment of the cumulus-oocyte complex from the follicle wall [70].

The optimal ORR group had a significantly higher mean number of MII (mature) oocytes which is not surprising. They also had a higher mean number of fertilized eggs, but this was not statistically significant. This is similar to findings from another study, where a low follicle-oocyte index while not significantly affecting outcomes, merely presented an opportunity for research to exploit new therapies to maximise oocyte yield and overlap prognosis [72]. Empty follicle syndrome, an extreme form of sub-optimal ORR where no oocytes at all are retrieved can be quite devastating and occurred in only six women. They are more likely due to the genuine form as all precautions are taken to prevent technical or drug-related causes. A lot of women in this environment advised on third-party treatments (due to advanced age or other features that may suggest poor ovarian response) still prefer to try with their eggs and some still succeed. Repeating triggers, dual triggers with another batch of HCG and repeating oocyte retrieval may be useful in such cases [73,74].

**Study limitations:** this study contributes to the dearth of fertility studies in this environment. However, it does have some limitations. This was a small study, and a timed convenient sample was used so results should be interpreted with some caution. Findings cannot be over-generalized as the sample is not representative of the whole population of Nigerian infertile women. Inter and intra-observer errors may have introduced some bias and subjectivity during follicular tracking (ultrasonography), partly reduced by defining optimal ORR as a range. Stimulation protocols and drugs used were not uniform, and not all possible confounders were controlled for. Better data keeping and follow-up would have helped track other clinical long-term outcomes, though this was not the focus of this study.

## Conclusion

ORR was significantly associated with BMI, pattern of menstruation (regularity, length and flow), trigger type, FSH levels, prolactin levels and number of MII oocytes. Having sub-optimal ORR however does not necessarily translate to significantly lower fertilization rates. More studies are however required, especially for relevance to other clinical outcomes. Studies to determine genetic causes of genuine EFS in this environment will also be useful. Communication with patients should continue after treatment irrespective of outcome.

### What is known about this topic

- *During ART some women are known to have poor responses;*
- *Follicles retrieved may differ from baseline antral follicular count but should correlate number seen at the trigger;*
- *Yet some women may even have empty follicle syndrome, which in genuine cases is difficult to treat*.

### What this study adds

- *Baseline data which is scarce in African settings;*
- *Additional potential marker, ORR, that can be used to monitor ART quality and outcomes;*
- *Reveals possible factors in this environment that may be associated with the number of follicles retrieved that may be explored further and modified to improve outcomes*.

## References

[1] Zegers-Hochschild F, Adamson GD, Mouzon J, Ishihara O, Mansour R, Nygren K (2009). The international committee for monitoring Assisted Reproductive Technology (ICMART) and the WHO revised glossary of ART terminology. Human Reproduction.

[2] Vander Borght M, Wyns C (2018). Fertility and infertility: Definition and epidemiology. Clin Biochem.

[3] Mascarenhas MN, Flaxman SR, Boerma T, Vanderpoel S, Stevens GA (2012). National, Regional, and Global Trends in Infertility Prevalence Since 1990: A Systematic Analysis of 277 Health Surveys. PLoS Med.

[4] Qiao J, Feng HL (2014). Assisted reproductive technology in China: compliance and non-compliance. Transl Pediatr.

[5] Lunenfeld B, Van Steirteghem A (2004). Infertility in the third millennium: implications for the individual, family and society: condensed meeting report from the Bertarelli Foundation´s second global conference. Hum Reprod Update.

[6] Nachtigall RD (2006). international disparities in access to infertility services. Fertil Steril.

[7] Boivin J, Bunting L, Collins JA, Nygren KG (2007). International estimates of infertility prevalence and treatment-seeking: Potential need and demand for infertility medical care. Hum Reprod.

[8] Chimbatata NB, Malimba C (2016). Infertility in sub-Saharan Africa: a Woman´s issue for how long? A qualitative review of literature. Open J Soc Sci.

[9] Adegbola O, Akindele MO (2013). The pattern and Challenges of infertility management in Lagos Nigeria. Afr Health Sci.

[10] Obuna JA, Ndukwe EO, Ugboma HA, Ejikeme BN, Ugboma EW (2012). Clinical Presentation of infertility in an outpatient clinic of a resource poor setting, South-East Nigeria. Int J Trop Dis Health.

[11] Panti AA, Sununu YT (2014). The Profile of Infertility in a teaching Hospital in North West Nigeria. Sahel Med J.

[12] Dattijo LM, Andreadis N, Aminu BM, Umar NI, Black KI (2016). The prevalence and clinical pattern of Infertility in Bauchi, Northern Nigeria. Trop J Obstet Gynaecol.

[13] Bushnik T, Cook JL, Yuzpe AA, Tough S, Collins J (2012). Estimating the prevalence of infertility in Canada. Hum Reprod.

[14] Dyer SJ (2007). The value of children in African countries-insights from studies on infertility. J Psychosom Obstet Gynaecol.

[15] Vayena E, Rowe JP, Peterson HB (2002). Assisted reproduction in developing countries: why should we care?. Fertil Steril.

[16] Gameiro S, Finnigan A (2017). Long-term adjustment to unmet parenthood goals following ART: a systematic review and meta-analysis. Hum Reprod Update.

[17] Odunvbun WO, Oziga DV, Oyeye LO, Ojeogwu CL (2018). Pattern of infertility among infertile couple in a secondary health facility in Delta State, South South Nigeria. Trop J Obstet Gynaecol.

[18] Otolorin EO, Ojengbede O, Falase AO (1987). Laparoscopic evaluation of the tuboperitoneal factor in infertile Nigerian women. Int J Gynaecol Obstet.

[19] Okonofua FE, Essen UI, Nimalaraj T (1989). Hysterosalpingography versus laparoscopy in tubal infertility: comparison based on findings at laparotomy. Int J Gynaecol Obstet.

[20] Otubu JA, Sagay AS, Dauda S (1990). Hysterosalpingogram, laparoscopy and hysteroscopy in the assessment of the infertile Nigerian women. East Afr Med J.

[21] Giwa-Osagie OF, Vayena E, Rowe PJ, Griffin PD (2002). ART in developing countries with particular reference to sub-Sahara Africa. Current practices and controversies in assisted reproduction.

[22] Ikechebelu JI, Adinma JI, Orie EF, Ikegwuonu SO (2003). High prevalence of male infertility in southeastern Nigeria. J Obstet Gynaecol.

[23] Araoye OM (2003). Epidemiology of infertility: social problems of the infertile couples. West Afr J Med.

[24] Ombelet W (2014). Is global access to infertility care realistic? The Walking Egg Project. Reprod Biomed Online.

[25] Te Velde E, Habbema D, Nieschlag E, Sobotka T, Burdorf A (2017). Ever growing demand for in vitro fertilization despite stable biological fertility-A European paradox. Eur J Obstet Gynecol Reprod Biol.

[26] Orhue A, Aziken M (2008). Experience with a comprehensive university hospital based infertility program in Nigeria. Int J Gynaecol Obstet.

[27] Kamphuis EI, Bhattacharya S, Van Der Veen F, Mol BW, Templeton A (2014). Are we overusing IVF?. BMJ.

[28] Okafor NI, Joe-Ikechebelu NN, Ikechebelu JI (2017). Perceptions of Infertility and In Vitro Fertilization Treatment among Married Couples in Anambra State, Nigeria. Afr J Reprod Health.

[29] Okwelogu IS, Azuike EC, Ikechebelu JI, Nnebue CK (2012). In vitro fertilization practice: Awareness and perceptions among women attending fertility clinics in Okija, Anambra state, Nigeria. Afr Med J.

[30] Orhue AA, Aziken ME, Osemwenkha AP, Ibadin KO, Odoma G (2012). In vitro fertilization at a public hospital in Nigeria. Int J Gynaecol Obstet.

[31] Edwards RG, Steptoe PC (1983). Current status of in-vitro fertilisation and Implantation of human embryos. Lancet.

[32] Hoult IJ, de Crespigny LC, O'Herlihy C, Speirs AL, Lopata A, Kellow G (1981). Ultrasound control of clomiphene/human chorionic gonadotropin stimulated cycles for oocyte recovery and in vitro fertilization. Fertil Steril.

[33] Wortham JW, Veeck LL, Witmyer J, Sandow BA, Jones HW (1983). Vital initiation of pregnancy (VIP) using human menopausal gonadotropin and human chorionic gonadotropin ovulation induction: phase II-1981. Fertil Steril.

[34] Inan MS, Al-Hassan S, Ozand P, Coskun S (2006). Transcriptional profiling of granulosa cells from a patient with recurrent empty follicle syndrome. Reprod Biomed Online.

[35] Revelli A, Carosso A, Grassi G, Gennarelli G, Canosa S, Benedetto C (2017). Empty follicle syndrome revisited: Definition, incidence, aetiology, early diagnosis and treatment. Reprod Biomed Online.

[36] Driscoll GL, Tyler JP, Knight DC, Cooke S, Kime L, Clark L (1998). Failure to collect oocytes in assisted reproductive technology: a retrospective study. Hum Reprod.

[37] Desai N, Austin C, AbdelHafez F, Goldfarb J, Falcone T. Evidence of (2009). “genuine” empty follicles in follicular aspirate: a case report. Hum Reprod.

[38] Coulam CB, Bustillo M, Schulman JD (1986). Empty follicle syndrome. Fertil Steril.

[39] Aktas M, Beckers NG, van Inzen WG, Verhoeff A, de Jong D (2005). Oocytes in the empty follicle: a controversial syndrome. Fertil Steril.

[40] Stevenson TL, Lashen H (2008). Empty follicle syndrome: the reality of a controversial syndrome, a systematic review. Fertil Steril.

[41] Tsuiki A, Rose B, Hung T (1988). Steroid profiles of follicular fluids from a patient with the empty follicle syndrome. Fertil Steril.

[42] Onalan G, Pabuccu R, Onalan R, Ceylaner S, Selam B (2003). Empty follicle syndrome in two sisters with three cycles: Case report. Hum Reprod.

[43] Vujisic S, Stipoljev F, Bauman R, Dmitrovic R, Jezek D (2005). Pericentric inversion of chromosome 2 in a patient with the empty follicle syndrome: case report. Hum Reprod.

[44] Lorusso F, Depalo R, Tsadilas S, Caradonna F, Di Gilio A, Capotorto MT (2005). Is the occurrence of the empty follicle syndrome a predictor that a subsequent stimulated cycle will be an unfavourable one?. Reproductive BioMedicine.

[45] Isik AZ, Vicdan K (2000). Borderline form of empty follicle syndrome: is it really an entity. Eur J Obstet Gynecol Reprod Biol.

[46] Nikolettos N, Asimakopoulos B, Simopoulou M, Al-Hasani S (2004). A borderline form of empty follicle syndrome. Case report. Clin Exp Obstet Gynecol.

[47] McLernon DJ, Maheshwari A, Lee AJ, Bhattacharya S (2016). Cumulative live birth rates after one or more complete cycles of IVF: a population-based study of linked cycle data from 178,898 women. Hum Reprod.

[48] Omokanye LO, Olatinwo AO, Durowade KA, Raji HO, Raji ST, Biliaminu SA, Ganiyu SA (2017). Determinants of utilization of assisted reproductive technology services in Ilorin, Nigeria. Journal of Medical Society.

[49] Mohammed-Durosinlorun A, Adze J, Bature S, Abubakar A, Mohammed C, Taingson M, Airede L (2019). Awareness, Acceptability and Affordability of Assisted Reproductive Technology among Infertile Women seen in a Tertiary Hospital in Northern Nigeria. Journal of Research in Basic and Clinical Sciences.

[50] Knopman JM, Grifo JA, Akiva P Novetsky, Meghan B Smith, Alan S Berkeley (2012). Is bigger better: The association between follicle size and livebirth rate following IVF?. Open Journal of Obstetrics and Gynecology.

[51] Nakamura M, Yamashita Y, Hayashi A, Saito N, Yu M, Hayashi M (2016). Analyzing the risk factors for a diminished oocyte retrieval rate under controlled ovarian stimulation. Reprod Med Biol.

[52] Wirleitner B, Okhowat J, Vištejnová L, Králíčková M, Karlíková M, Vanderzwalmen P (2018). Relationship between follicular volume and oocyte competence, blastocyst development and live-birth rate: optimal follicle size for oocyte retrieval. Ultrasound Obstet Gynecol.

[53] Wada R, Hati SS, Ofoli JNT, Wada I (2016). Service Quality Domains Impelling Patient´s Return Intentions in Nisa Premier Hospital Abuja. International Business and Management.

[54] Jie Q, Huai L (2014). Assisted reproductive technology in China: Compliance and non compliance. Transl Pediatr.

[55] Steward RG, Lan L, Shah AA, Yeh JS, Price TM, Goldfarb JM (2014). Oocyte number as a predictor for ovarian hyperstimulation syndrome and live birth: an analysis of 256,381 in vitro fertilization cycles. Fertil Steril.

[56] Magnusson Å, Källen K, Thurin-Kjellberg A, Bergh C (2018). The number of oocytes retrieved during IVF: a balance between efficacy and safety. Hum Reprod.

[57] Christensen MW, Ingerslev HJ, Degn B, Kesmodel US (2016). Effect of Female Body Mass Index on Oocyte Quantity in Fertility Treatments (IVF): Treatment Cycle Number Is a Possible Effect Modifier. A Register-Based Cohort Study. PLoS One.

[58] Obose R, Osaikhuwuomwan J, Aziken M (2019). Body mass index and Ovarian response in an In-Vitro Fertilization Cycle. Rwanda Journal of Medicine and Health Sciences.

[59] Small CM, Manatunga AK, Klein M, Feigelson HS, Dominguez CE, McChesney R (2006). Menstrual Cycle Characteristics: Associations with Fertility and Spontaneous Abortion. Epidemiology.

[60] Ting Y, Di W, Yurong C, Jun Z (2021). Association Between Menstrual Patterns and Adverse Pregnancy Outcomes in Patients With Polycystic Ovary Syndrome. Front Endocrinol (Lausanne).

[61] Jehan S, Iram Z, Syed S (2020). Empty follicle syndrome: Frequency and probable causes in Pakistani population. J Pak Med Assoc.

[62] Huang LN, Jun SH, Drubach N, Dahan MH (2015). Predictors of In Vitro Fertilization Outcomes in Women with Highest Follicle-Stimulating Hormone Levels ≥12 IU/L: A Prospective Cohort Study. PLoS ONE.

[63] Azziz R, Adashi EY (2016). Stein and Leventhal: 80 years on. Am J Obstet Gynecol.

[64] Mendoza C, Cremades N, Ruiz-Requena E, Martinez F, Ortega E, Bernabeu S (1999). Relationship between fertilization results after intracytoplasmic sperm injection, and intrafollicular steroid, pituitary hormone and cytokine concentrations. Hum Reprod.

[65] Zhang Y, Guo X, Guo L, Chang HM, Shu J, Leung PCK (2021). Outcomes comparison of IVF/ICSI among different trigger methods for final oocyte maturation: A systematic review and meta-analysis. The FASEB J.

[66] Juan C Castillo, Juan Garcia-Velasco, Peter Humaidan (2012). Empty follicle syndrome after GnRHa triggering versus hCG triggering in COS. J Assist Reprod Genet.

[67] Beck-Fruchter R, Weiss A, Lavee M, Geslevich Y, Shalev E (2012). Empty follicle syndrome: successful treatment in a recurrent case and review of the literature. Human Reproduction.

[68] Lamb JD, Shen S, McCulloch C, Jalalian L, Cedars MI, Rosen MP (2011). Follicle-stimulating hormone administered at the time of human chorionic gonadotropin trigger improves oocyte developmental competence in in vitro fertilization cycles: a randomized, double-blind, placebo-controlled trial. Fertil Steril.

[69] Ding N, Liu X, Jian Q, Liang Z, Wang F (2017). Dual trigger of final oocyte maturation with a combination of GnRH agonist and hCG versus a hCG alone trigger in GnRH antagonist cycle for in vitro fertilization: A Systematic Review and Meta-analysis. Eur J Obstet Gynecol Reprod Biol.

[70] Abdalla IH, Ah-Moye M, Brisden P, Howe DL, Okonofus F, Craft I (1987). The effect of the dose of human chorionic gonadotropin and the type of gonadotropin stimulation on oocyte recovery rates in an in vitro fertilization program. Fertil Steril.

[71] van Loenen ACD, Huirne JAF, Schats R, Hompes PGA, Lambalk CB (2002). Gonadotrophin-releasing hormone Agonist, antagonist and assisted reproduction. Semin Reprod Med.

[72] Carosso AR, van Eekelen R, Revelli A, Canosa S, Mercaldo N, Benedetto C (2022). Women in Advanced Reproductive Age: Are the Follicular Output Rate, the Follicle-Oocyte Index and the Ovarian Sensitivity Index Predictors of Live Birth in an IVF Cycle. J Clin Med.

[73] Ndukwe G, Thornton S, Fishel S, Dowell K, Al-Hassan S, Hunter A (1996). Predicting empty follicle syndrome. Fertil Steril.

[74] Ndukwe G, Thornton S, Fishel S, Dowell K, Aloum M, Green S (1997). 'Curing' empty follicle syndrome. Hum Reprod.

